# Dynamic cellular complexity of anoxygenic phototrophic sulfur bacteria in the chemocline of meromictic Lake Cadagno

**DOI:** 10.1371/journal.pone.0189510

**Published:** 2017-12-15

**Authors:** Francesco Danza, Nicola Storelli, Samuele Roman, Samuel Lüdin, Mauro Tonolla

**Affiliations:** 1 Laboratory of Applied Microbiology (LMA), Department for Environmental Constructions and Design (DACD), University of Applied Sciences and Arts of Southern Switzerland (SUPSI), via Mirasole 22a, Bellinzona, Switzerland; 2 Microbiology Unit, Department of Botany and Plant Biology, University of Geneva, Geneva, Switzerland; 3 Alpine Biology Center Foundation, via Mirasole 22a, Bellinzona, Switzerland; 4 Federal Office for Civil Protection, Spiez Laboratory, Biology Division, Spiez, Switzerland; Universidade Nova de Lisboa, PORTUGAL

## Abstract

The meromictic Lake Cadagno is characterized by a compact chemocline with high concentrations of anoxygenic phototrophic purple sulfur bacteria (PSB) and green sulfur bacteria (GSB). The co-occurrence of phylogenetically distant bacterial groups such as PSB and GSB in the same ecological niche, makes the chemocline of Lake Cadagno an ideal system for studying the conditions and consequences of coexistence of photosynthetic bacteria populations. In this study, we applied flow cytometry (FCM) as a fast tool to identify metabolic changes due to the production and consumption of inclusion bodies such as sulfur globules (SGBs), and follow population dynamics of closely related anoxygenic photosynthetic sulfur bacteria in their natural environment. Large-celled PSB *Chromatium okenii* and GSB *Chlorobium* populations were reliably separated and identified due to differences in auto-fluorescence and cell size. Moreover, we showed that these dominant taxa share the same ecological niche over seasonal periods. Taking advantage of FCM detection of dynamic cellular complexity variation during phases of photosynthetic activity, we identified an unexpected alternation in PSB versus GSB metabolic activity, indicating dynamic interspecific interactions between these two populations.

## Introduction

Photosynthesis converts light energy to chemical energy stored in the form of organic carbon compounds through the fixation of inorganic CO_2_. This process enables autotrophic organisms to form the basis of food webs in most ecosystems as primary producers. Global estimates suggest that bacteria are responsible for up to 98% of organic carbon produced on Earth [1], but they are also key mediators in most other biogeochemical cycles [2].

Other than oxygenic photosynthesis occurring in plants, algae and aerobic microorganisms, anoxygenic photosynthesis is an important ecosystem process driven by anaerobic photosynthetic microorganisms, which play a dominant role in CO_2_-fixation in anaerobic environments [3,4]. In contrast to water photolysis as electron source, anoxygenic photosynthesis uses electron donors such as hydrogen (H_2_), hydrogen sulfide (HS^-^), thiosulfate (S_2_O_3_^2-^), sulfur (S^0^) and reduced iron (Fe^2+^).

Photosynthetic sulfur bacteria are the dominant anoxygenic phototrophs in natural anoxic environments, and are divided into two phylogenetic distant groups: purple sulfur bacteria (PSB) belonging to the order *Chromatiales* and green sulfur bacteria (GSB) belonging to the orders *Chlorobiales* [5]. The most abundant family of GSB is *Chlorobiaceae*, which are usually strict photolithotrophic and which deposit sulfur globules (SGBs) extracellularly during sulfide oxidation [6–9]. In contrast, PSB of the family *Chromatiaceae*, which are capable of both photoautotrophy and photoheterotrophy, accumulate SGBs inside the cell, inducing a change in cell morphology [10].

Anoxygenic photosynthetic sulfur bacteria grow by photolithoautotrophic oxidation of reduced sulfur compounds in environments with opposite gradients of light and sulfide. Therefore, their development in aquatic environments requires the existence of an illuminated anoxic compartment that may develop especially in stratified systems. Shallow meromictic lakes are thus ideal environments for the development of communities of photosynthetic sulfur bacteria [11].

The alpine meromictic Lake Cadagno located in the southern part of Switzerland has been intensely investigated during the last decades [12–17]. Its chemocline, typically at a depth of about 12 meters, separates the oxic (low salt concentrations, lower density) mixolimnion from the anoxic (high salt concentrations, higher density) monimolimnion. The chemocline, at the transition between oxic and anoxic water and with opposite gradients of sulfide and light intensity, stimulates the growth of a dense community of anoxic phototrophic sulfur bacteria, especially during summer period [18–20]. PSB of the genera *Chromatium*, *Lamprocystis*, *Thiocystis* and *Thiodictyon* and GSB of the genus *Chlorobium* are well characterized and their role for the ecology of the lake was elucidated in earlier studies [15,16,21].

The co-occurrence of phylogenetically distant bacterial groups such as PSB and GSB in the same confined niche with limited nutrients makes the Lake Cadagno chemocline an ideal natural system for studying the coexistence of photosynthetic bacterial populations [11]. The advent of GSB in Lake Cadagno in the year 2000 increased the density of the photosynthetic community in the chemocline without affecting the density of the PSB populations themselves [20,22,23]. Possible conditions that could favor the coexistence of two competing organisms such as PSB and GSB were proposed and include complementary relationships on mutual substrates. However, besides investigations on contributions of single bacterial populations from the chemocline of Lake Cadagno to inorganic carbon fixation [15,21,24], the mechanisms underlying their coexistence are unknown [11]. Indeed the biological mechanisms modulating and regulating their metabolic activity and thus permitting coexistence in this particular habitat are still poorly understood. Elucidating the strategies adopted by the involved species may shed light on these mechanisms.

During the last decades, flow cytometry (FCM) became an essential tool in the field of aquatic microbiology, providing opportunities for microbial analysis at both the community and single-cell level [25–28]. In photosynthetic microorganisms, cellular fluorophores such as chlorophyll and phycobilins respond with emission of specific wavelengths (> 640 nm emission wavelength with 488 nm exciting laser for chlorophyll, and 650 nm emission wavelength with 640 nm exciting laser for phycobilins) to FCM laser-beam excitation. Therefore, photosynthetic organisms can be directly discriminated without the addition of dyes or probes [29]. A study in the karstic meromictic Lake Vilar (Spain) showed the possibility to rapidly identify and count PSB and GSB populations [30].

In addition to estimates of population density, FCM allows rapid characterization of bacterial cell states. Growth, death, replication, cell division, metabolism and cell-surface phenomena can be observed in real-time, providing a tool to investigate environmental effects on cellular physiology [29]. FCM can discriminate distinct fractions of bacteria within mixed assemblages and thus allows monitoring single populations within heterogeneous bacterial communities. Light scattering parameters were applied for elucidating bacterial activity based on size and nucleic acid content in planktonic bacteria in their habitat [31]. Forward scatter (FSC) and sideward scatter (SSC) are the parameters commonly used to describe bacterial population structure, with FSC correlating to cell size and SSC to cellular density or granularity. Consequently, variations in the latter may be used as indicators of cellular activity and physiological changes in bacterial populations [32]. In particular, cellular inclusion bodies (i.e SGBs, glycogen, polyhydroxybutyrate [PHB], proteins) may affect the scattered light (measured as SSC) without changes in cell size (measured as FSC) [30,33].

The formation of storage inclusion bodies affects cellular complexity and morphology in phototrophic sulfur bacteria [34].The FCM SSC signal generated by phototrophic sulfur bacteria was described to directly depend on the photosynthetically driven storage of intracellular SGBs [30]. However, at the present these observations conducted under laboratory conditions were never verified *in situ*.

In contrast to previous studies investigating phototrophic bacterial population dynamics in the chemocline of Lake Cadagno by FISH [19,22,23], we applied FCM for the rapid identification and quantification of the two most abundant populations of phototrophic sulfur bacteria; the large-celled PSB *Chromatium okenii* and the GSB *Chlorobium* spp. Moreover, we evaluated the metabolic activity *in vitro* and *in situ* through variations in SSC related to changes in cellular complexity due to the formation or consumption of intracellular inclusion bodies.

## Material and methods

### Cultivation of phototrophic purple and green sulfur bacteria

PSB were grown in Pfennig’s medium I [35], whereas GSB were grown in Pfennig’s medium II (Biebl & Pfennig, 1979), both of which contain 0.25 g of KH_2_PO_4_ L^-1^, 0.34 g of NH_4_Cl L^-1^, 0.5 g of MgSO_4_ x 7H_2_OL^-1^, 0.25 g of CaCl_2_x2H_2_OL^-1^, 0.34 g of KCl L^-1^, 1.5 g of NaHCO_3_ L^-1^, 0.02 mg of vitamin B12 L^-1^ and 0.5 mL of trace element solution SL12 L^-1^ for PSB and SL10 for GSB. The media were prepared in 2-L bottles using a flushing gas composition of 80% N_2_ and 20% CO_2_ according to [36] and were reduced by the addition of 1.10 mM Na_2_S × 9H_2_O and adjusted to a pH of 7.0. All cultures were incubated at room temperature (20–23°C) and subjected to a light/dark photoperiod of 12 h with a light intensity of 5 μE m^-2^ s^-1^ (measured with LI-193SA spherical quantum sensor, LI-COR Ltd, Lincoln, NE) with incandescent 60 W bulbs emitting the entire white spectrum.

In particular, these conditions were used for the cultivation of small-celled PSB *Candidatus* “Thiodictyon syntrophicum” strain Cad16^T^, GSB *Chlorobium phaeobacteroides* and for enrichment of large-celled PSB *Chromatium okenii*.

### Sampling site and water sampling

Lake Cadagno is a meromictic alpine lake located in the Piora Valley at 1921 meters above sea level in southern Switzerland. The sampling season in Lake Cadagno started in June (after ice-melt) and ended in October (first snow). Samples used in the present study were obtained in 2015 and 2016 from a platform moored above the deepest point of the lake (46.55087° N, 8.71153° E, depth approximately 21 m). Water for chemical and biological analysis was sampled with a 1 L Niskin bottle. For sampling at high vertical resolutionwater was pumped to the surface through a Tygon-tube (20 m long, inner diameter 6.5 mm, volume 0.66 L) at a flow rate of 1L min^-1^ using a peristatic pump (Cole-Parmer Instrument Co., USA, Universal Electric Co., USA). Samples were stored in 15 mL falcon tubes in the dark and analysed for microbiological parameters no later than 1 hour after sampling.

The University of Geneva has an official permission from the Government of Canton Ticino for the scientific works on Lake Cadagno.

### Measurement of physico-chemical parameters of Lake Cadagno

Turbidity (NTU) in the water column was measured with a turbidity sensor (ECO NTU, WET Labs, Sea-Bird, Bellevue, WA, USA) of a Sea-Bird CTD (conductivity, temperature, depth; SBE 19plus V2, Sea-Bird, Bellevue, WA, USA). The sensor measured backscattered light emitted at 700 nm with a sensitivity of 0.02 NTU. Oxygen (mg L^-1^) was measured with a membrane-based probe (OxyGuard, Ocean Probe, Farum, Denmark) mounted on another multiparameter probe (CTM281, Sea & Sun Technology, Trappenkamp, Germany) measuring at 2.4Hz.

Sulfide was measured from 12 mL samples that were immediately transferred to screw capped tubes containing 0.8 mL of 4% zinc acetate solution. These samples were stored in the dark and analyzed colorimetrically using a Spectroquant kit (Merck, Schaffhausen, Switzerland).

### Flow cytometry analysis of Lake Cadagno water samples

For the detection and quantification of phototrophic sulfur bacteria in the water column, FCM analysis was conducted measuring chlorophyll-like autofluorescence particle events. A BD Accuri C6 cytometer (Becton Dickinson, San José, CA, USA) equipped with two lasers (488 nm, 680 nm), two scatter detectors, and four fluorescence detectors (laser 488nm: FL1 = 533/30, FL2 = 585/40, FL3 = 670; laser 640 nm: FL4 = 670) was used to measure two parameters for event characterization: forward scatter (FSC) which correlates with particle size, and 90° light scatter (SSC), which relates to internal granularity of the particles.

For the identification of photosynthetic bacteria a first forward scatter threshold of FSC-H 10’000 was applied, which excludes debris and abiotic particles. Subsequently a FL3-A > 1’100 threshold was applied using FL3 (red fluorescence), to select cells emitting autofluorescence due to chlorophyll and bacteriochlorophyll. Sample analysis was limited to 50 μL at a flow rate of 66 μL min^-1^ and samples were diluted if necessary to achieve no more than 1’000 events per mL.

Green sulfur (GSB) and purple sulfur bacteria (PSB) colonizing the chemocline of Lake Cadagno were distinguished through FCM based on morphological characters. Among PSB,large-celled *C*. *okenii* (~ 7 μm) and GSB *Chlorobium* spp. (~ 0.8 μm) were clearly separated from the other populations in SSC vs FSC dot-plots. GSB *Chlorobium* spp. and PSB *C*. *okenii* gating permitted their respective counts in the anaerobic phototrophic community. Confirmation of taxon-specific FCM signals in chemocline samples and definition of specific gates was based on FCM measurements of PSB *Candidatus* “T. syntrophicum” strain Cad16^T^, GSB *C*. *phaeobacteroides and C*. *clathratiforme* and PSB *C*. *okenii* from pure or enrichment cultures.

### Flow cytometry characterization of PSB and GSB responses to sulfide oxidation

The response of phototrophic sulfur bacteria to light-dependent sulfide oxidation and subsequent accumulation of SGBs was tested for PSB *C*. *okenii*, PSB model strain *Candidatus* “T. syntrophicum” Cad16^T^ and GSB *C*. *pheobacteroides*.

For PSB *C*. *okenii*, 60 mL enrichment from the Lake Cadagno chemocline (~ 6 × 10^5^ mL^-1^) were pulsed with H_2_S solution to a final concentration of 0.45 mM and incubated under irradiation of 6 μE m^-2^ s^-1^ (measured with LI-193SA spherical quantum sensor, LI-COR Ltd, Lincoln, NE) with incandescent 60 W bulbs. As negative control, replicates were incubated in the dark in absence of light.

Similarly, for GSB *C*. *pheobacteroides*, 60 mL culture (~10^8^ mL^-1^) were pulsed with H_2_S solution to a final concentration of 0.25 mM and incubated under irradiation (6 μE m^-2^ s^-1^)with incandescent 60 W bulbs again with negative controls kept in the dark.

For PSB, the *Candidatus* “T. syntrophicum” strain Cad16^T^ (10^8^ cells mL^-1^) was centrifuged for 20 minutes at 8’000 rpm, resuspended and incubated overnight in 80 mL basal medium (0.25 g/L KH_2_PO_4_, 0.34 g/L NH_4_Cl, 0.34 g/L KCl, 0.5 g/L MgSO_4_ × 7H_2_O, 0.25 g/L CaCl_2_ × 2H_2_O). One mL of starved PSB Cad16^T^ suspension was then inoculated in 80 mL Pfennig medium with H_2_S concentration of 0.5 mM and incubated under irradiation of 6 μE m^-2^ s^-1^ with incandescent 60 W bulbs. As a negative control, a batch of 0.5 mM H_2_S was incubated in the dark in absence of light

All conditions were measured in triplicates. Samples were collected through the rubber stoppers using a N_2_-flushed syringe every two hours for FCM analysis, measurement of H_2_S concentration and microscopical detection of inclusion bodies.

Median values of forward scatter (FSC) and sideward scatter (SSC) were used to characterize cellular structural variation in response to H_2_S oxidation. For PSB *C*. *okenii* relative total nucleic acids content was estimated through staining with SYBR green I (Molecular Probes, Eugene, Oreg.). Samples were stained with 1:10’000 (vol/vol) SYBR green I, incubated 13 minutes at 37°C in the dark. Histogram of counts vs green fluorescence (FL1 > 1’100) and gating on *C*. *okenii* population allowed detecting changes in signal intensity due to variation of nucleic acid content.

PSB intracellular SGBs were determined by light microscopy with a 1000x magnification for at least 20 fields at each sampling date.

### Statistical correlation of PSB *C*. *okenii* and GSB *Chlorobium* spp. in Lake Cadagno chemocline

To investigate metabolic activity pattern of the dominant populations, SSC values of *C*. *okenii* and *Chlorobium* spp. were measured every 4 hours over two days, which allowed quantifying variations in cellular complexity. Specific FCM gates were defined for PSB *C*. *okenii* and GSB *Chlorobium* and used as constant signature for their identification. 95%-confidence intervals of FCM SSC were measured for all events contained in the specific gate of the respective population. Measurements were done at maximal community density of the anoxygenic photosynthetic sulfur bacteria.

### Fluorescence *in situ* hybridization

Anoxygenic photosynthetic purple and green sulfur bacteria and sulfate-reducing bacteria in the water column of Lake Cadagno were identified and quantified using fluorescent *in situ* hybridization (FISH) with species- specific Cy3-labeled oligonucleotides (S1 Tab) in 1-μL aliquots of paraformaldehyde-fixed water samples (n = 3) spotted onto gelatin-coated slides [0.1% gelatin, 0.01% KCr(SO_4_)_2_] [37]. Hybridizations were performed as described in [38]. The slides were treated with Citifluor AF1 (Citifluor Ltd., London, UK) and examined by epifluorescence microscopy using filter sets F31 (AHF Analysentechnik, Tübingen, Germany; D360/40, 400DCLP, and D460/50 for DAPI) and F41 (AHF Analysentechnik; HQ535/50, Q565LP, and HQ610/75 for Cy3). Microorganisms were counted at a 1000-fold magnification in 40 fields of 0.01 mm^2^ each [39].

## Results

### Dynamic response to sulfide oxidation in PSB and GSB

**Fig 1:**
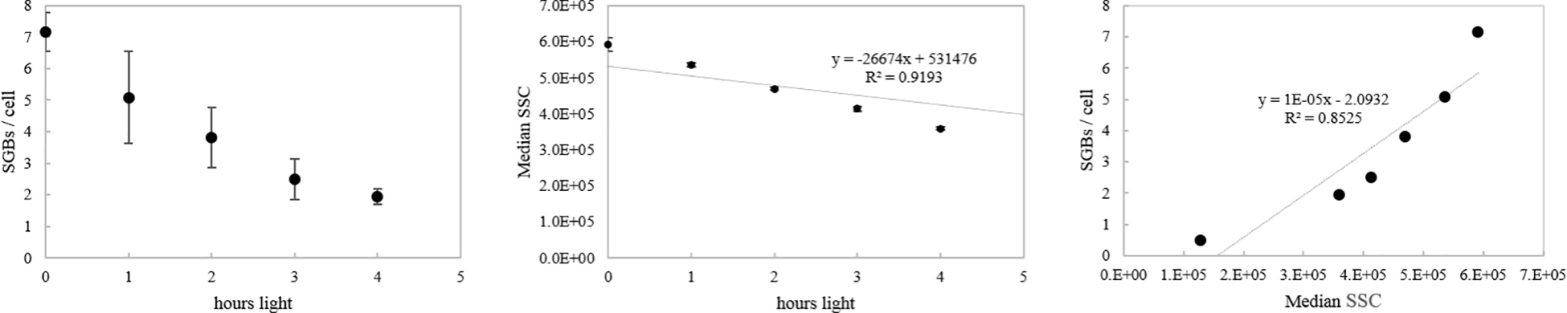
Correlation between SGBs per cell and FCM median SSC for anoxygenic PSB *C*. *okenii*. (A)Intracellular sulfur globules (SGBs) consumption per cell in function of light hours irradiation. (B) Flow cytometry median sideward scatter (SSC) decrease in function of light hours irradiation. (C) Correlation between number of SGBs per cell and median SSC in PSB *C*. *okenii*.

The fate of intracellular SGBs in the PSB family is related to light-dependent phototrophic oxidation of H_2_S present in the environment. Indeed, the amount of SGBs in large-celled PSB *C*. *okenii* decreased over time under continuous light irradiation in microcosm without addition of extra sulfide (Fig 1). Moreover, the consumption of SGBs coincided with the decrease in FCM SSC signal. Consequently, a linear correlation between those two parameters emerged.

**Fig 2:**
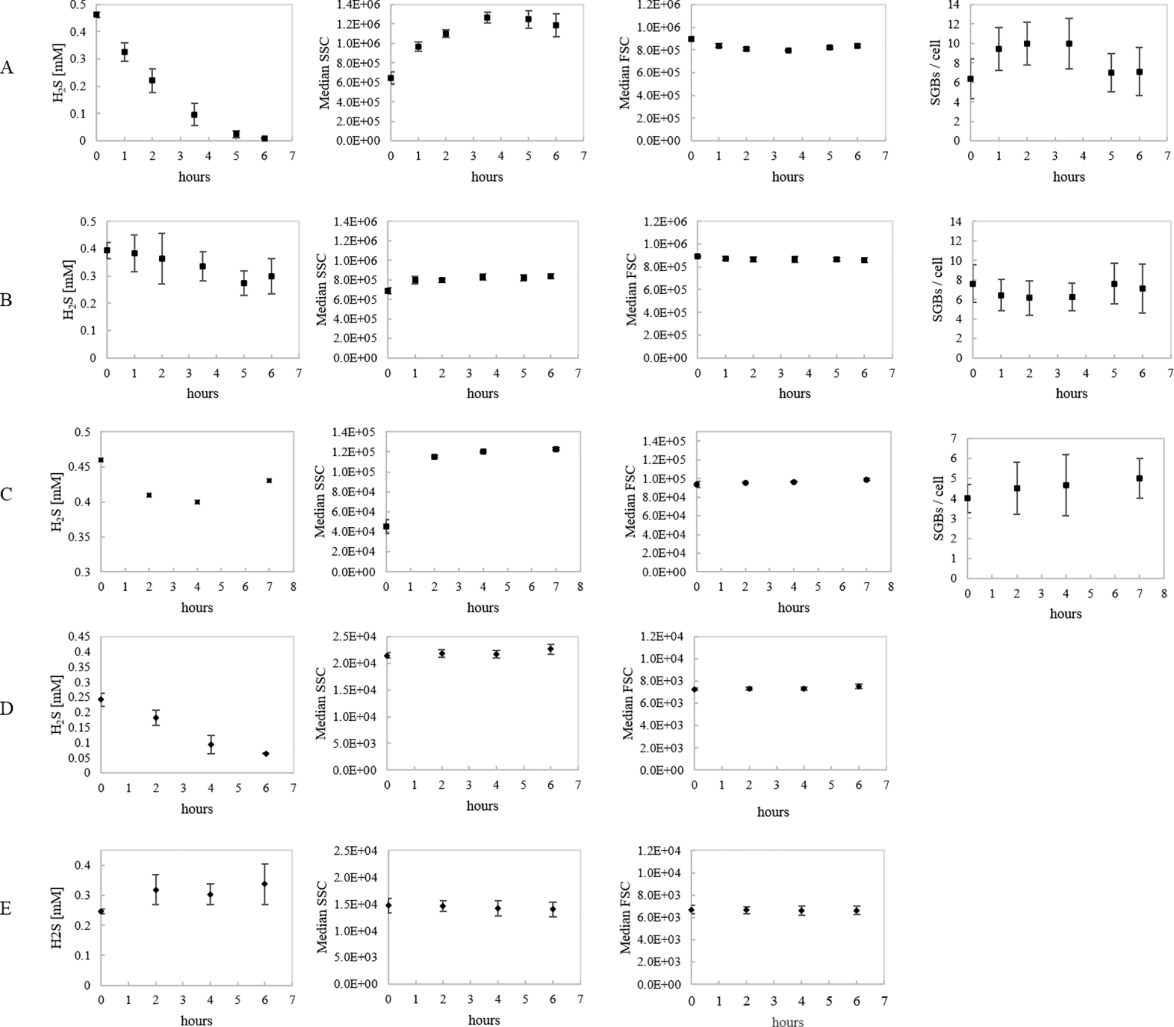
Flow cytometry dynamic response to sulfide oxidation in anoxygenic photosynthetic sulfur bacteria. H_2_S concentration [mM], median SSC, median FSC and SGBs cell^-1^ for (A) PSB *C*. *okenii* under light incubation, (B) PSB *C*. *okenii* under dark incubation, (C) PSB Candidatus “T. syntrophicum” strain Cad16^T^ under light incubation, (D) GSB *C*. *phaeobacteroides*. under light incubation, (E) GSB *C*. *phaeobacteroides* under dark incubation. Starting concentration was 0.45 mM for PSB and 0.25 mM for GSB. Error bars represent standard deviation (N = 3). If no error bars are shown, SD was smaller than the symbols used.

**Fig 3:**
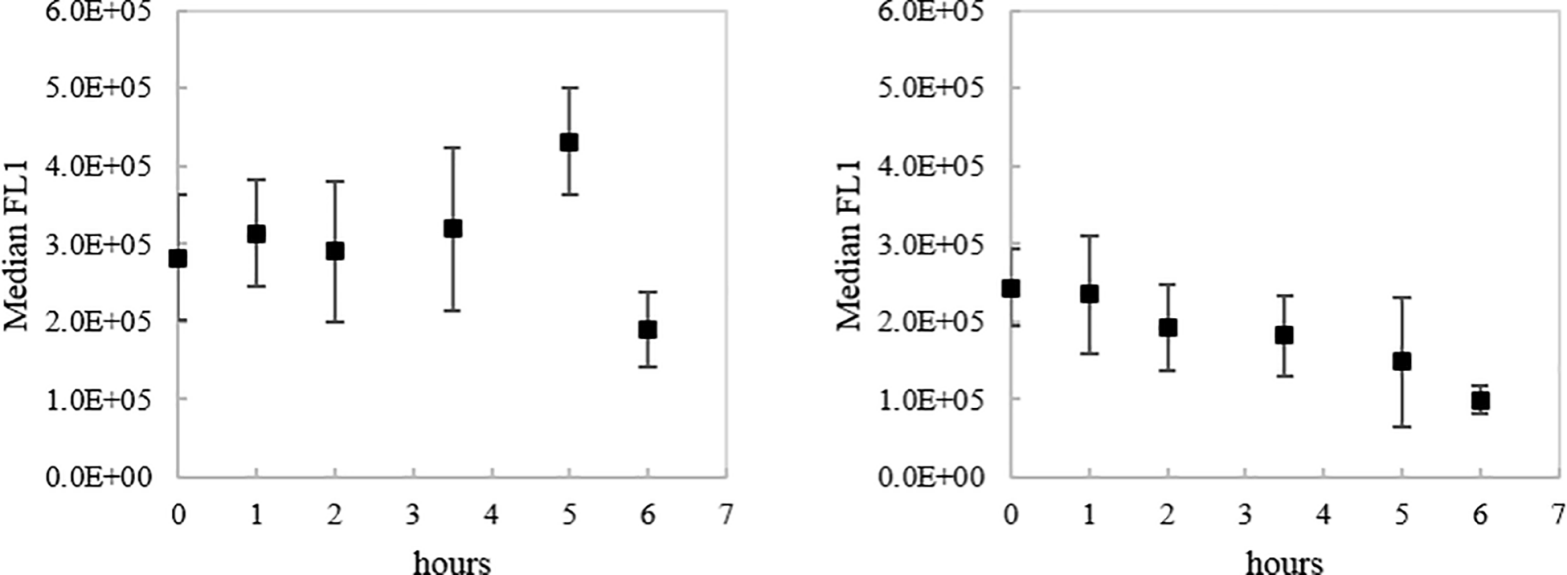
Nucleic acid intensity variation determined by FCM SYBR green staining in PSB *C*. *okenii*. Median FL1 signal intensity variation in function of light for PSB *C*. *okenii* under light (left) and dark (right) incubation after SYBR green staining for relative detection of double or single stranded DNA or RNA.

Under irradiation the H_2_S-oxidation rate of PSB *C*. *okenii* correlated with variations in SSC signal intensity and number of intracellular SGBs (Fig 2A). With a starting concentration of 0.45 mM H_2_S, *C*. *okenii* showed an oxidation rate of 2 × 10^−3^ nM h^-1^ cell^-1^. Sulfide consumption correlated with an increase of SSC (Δ ~ 6.1 × 10^5^). Subsequently, the gradual decrease in SSC occurring after 5 hours of irradiation followed the H_2_S depletion, probably due to the use of intracellular SGBs as electrons donors in photosynthesis. In contrast, the FCM forward scatter (FSC) signal did not vary significantly during light-dependent H_2_S oxidation. In the control microcosms kept in the dark (Fig 2B), H_2_S was not consumed and no variation in SSC, FSC and intracellular number of SGBs were observed. A similar behavior was observed for another PSB, the small-celled PSB *Candidatus* “Thiodictyon syntrophicum” strain Cad16^T^ (Fig 2C). SYBR green staining of PSB *C*. *okenii* showed that nucleic acid content followed parallel trends to FCM SSC during light and dark incubation (Fig 3).

In contrast to the dynamic variation observed for PSB *C*. *okenii*, light-dependent H_2_S oxidation did not affect SSC or FSC of GSB *C*. *phaeobacteroides*. (Fig 2D and 2E).

### Vertical distribution of phototrophic microorganisms in the water column of Lake Cadagno as determined by FCM

**Fig 4:**
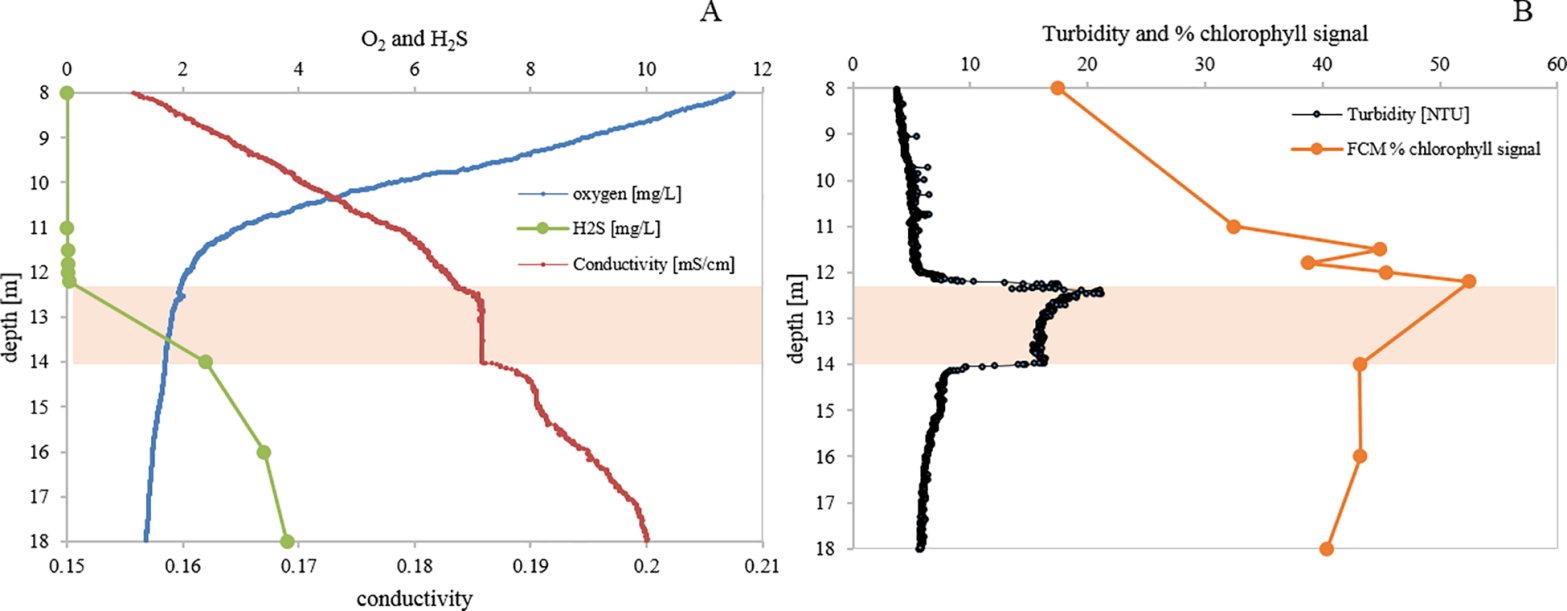
Physico-chemical and biological profiles of Lake Cadagno water column. A) Oxygen [mg L^-1^], H_2_S [mg L^-1^], conductivity [mS cm^-1^]. (B) Flow cytometry detected percentage of microbial chlorophyll and bacteriochlorophyll (% red fluorescence, FL3 positive-signal) and turbidity profile [NTU]. Orange shading highlight the chemocline layer on the sampling day (12 July 2016).

The Lake Cadagno chemocline is characterized by the decline of oxygen concentrations, the increase of sulfide and a homogeneous conductivity zone (Fig 4A). Analysis of the water column using a red autofluorescence (670 nm) signal confirmed the presence of photosynthetic microorganisms at high concentrations in the chemocline, coinciding with a turbidity peak (Fig 4B). In the upper part of the oxygenic layer (0–10 meters depth), the relative density of autofluorescent photosynthetic microorganisms was less than 20% (compared to total FCM events), whereas just above the chemocline (10–12 meters depth), the relative density doubled to 40% correlating with the presence of aerobic cyanobacteria and phytoplankton [40]. At 12 meters depth, 50% of total FCM events corresponded to chlorophyll-containing microorganisms. In the lower chemocline (i.e. between 12–14 meters) maximal turbidity correlated with the maximum of FCM red fluorescence signal and was associated with a peak in density of anoxygenic photoautotrophic sulfur bacteria. In the lower anoxygenic layer (between 14 and 18 meters) the relative density of autofluorescent phototrophic microorganisms was still high at approximately 40%.

### Flow-cytometry discrimination of dominant phototrophic sulfur bacteria in the chemocline of Lake Cadagno

**Fig 5:**
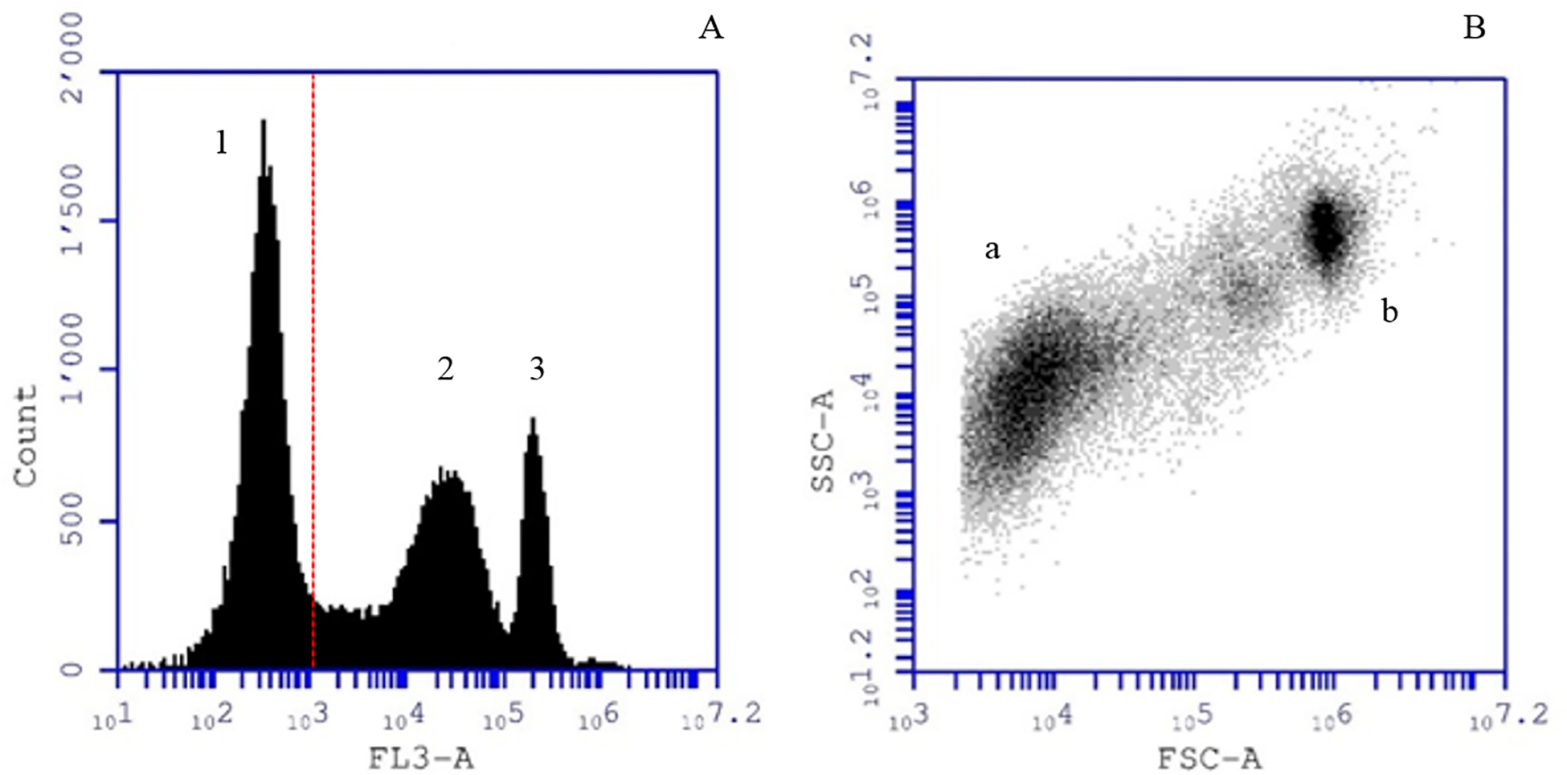
Flow-cytometry identification of phototrophic populations in the chemocline of Lake Cadagno. Lake Cadagno sample at 12.2 meters depth, 12 July 2016. (A) Histogram count versus red fluorescence (logarithmic FL3-A). Red fluorescence (FL3-A > 1’100) indicates the presence of chlorophyll-pigmented cells. (B) Scatter plot SSC versus FSC highlights two photosynthetic bacterial populations. Population *a* has a similar FCM-scatter signature as GSB *Chlorobium* spp., whereas population *b* has a similar FCM-scatter signature as PSB *C*. *okenii* as measured from pure cultures.

The CTD analysis of the water column suggested a maximal concentration of phototrophic sulfur bacteria at 12.2 m depth (12 July 2016, 9AM). At this depth, the red fluorescence histogram of FCM showed three distinct peaks (Fig 5A). Chlorophyll-positive autofluorescent FL3 signals greater than 1’100 correlated with the presence of anoxygenic photosynthetic sulfur bacteria. Peak 2 (FL3 = 27’701 in Fig 5A) and peak 3 (FL3 = 221’619 in Fig 5A) represented 32.4% and 14.7% of all FCM counts, respectively. This difference in FL3 intensity corresponded to two photosynthetic populations with contrasting pigment composition. Conversion of FL3 peaks 2 and 3 to a SSC vs FSC scatterplot revealed these two distinct photosynthetic populations (Fig 5B).

At 12.2 meters depth, population A (corresponding to peak 2 in Fig 5B) had an approximate cell size of 1.5 μm (median FSC = 7’181), and represented 58.6% of all autofluorescent bacterial cells. This population showed the same scattering signature as our pure culture of phototrophic GSB *Chlorobium* spp. isolated from Lake Cadagno (S1 Fig). Additionally, population B (highlighted as peak 3, in Fig 5B) had an approximate cell size of 7μm (median FSC = 910’106), representing 22% of all photosynthetic events. This signal corresponded to a signature measured from large-celled PSB *C*. *okenii* under laboratory conditions (S2 Fig).

**Fig 6:**
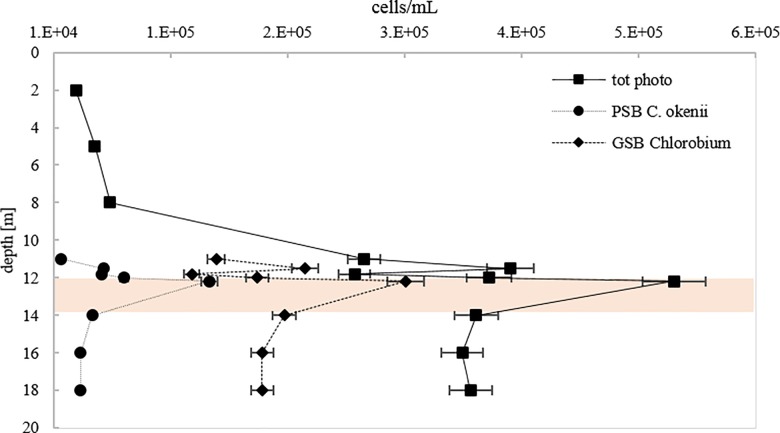
Flow cytometry quantification of phototrophic cells in Lake Cadagno water column. Total photosynthetic cells, PSB *C*. *okenii*, GSB *Chlorobium* spp. vertical profile. Orange shading highlight chemocline layer (9:00 AM, 12 July 2016).

The distinct scattering signature of large-celled PSB *C*. *okenii* vs GSB *Chlorobium* spp. and a gating strategy allowed separate analyses of the two populations and in turn estimates of their vertical distribution (Fig 6). Maximal population density for both PSB *C*. *okenii* (1.4 × 10^5^ cells mL^-1^) and GSB *Chlorobium* spp. (3.1 × 10^5^ cells mL^-1^) was at 12.2 meters depth.

Flow-cytometry quantification and fluorescence *in situ* hybridization (FISH) provided population density estimates of PSB *C*. *okenii* and GSB *Chlorobium* spp. in the same order of log-magnitude (i.e. 10^4^ for *C*. *okenii*, 10^5^ for *Chlorobium* spp.; Table 1).

**Table 1:** Comparison between FISH (± standard error) and FCM (± 5%, maximal machine error) quantification of PSB *C*. *okenii* and GSB *Chlorobium* spp. in the chemocline of Lake Cadagno (12 July 2016).

	PSB *C*. *okenii* (10^4^ mL^-1^)		GSB *Chlorobium* spp. (10^5^ mL^-1^)	
Depth (m)	FISH	FCM	FISH	FCM
11.0	0.67 ± 0.15	1.6 ± 0.08	1.37 ± 0.16	1.49 ± 0.075
11.5	3.2 ± 0.4	5.3 ± 0.27	3.74 ± 0.35	2.25 ± 0.11
11.8	4.2 ± 0.4	5.1 ± 0.26	3.22 ± 0.30	1.28 ± 0.06
12.0	4.3 ± 0.4	7.0 ± 0.35	4.81 ± 0.29	1.84 ± 0.092
12.2	7.8 ± 0.6	14.3 ± 0.71	4.39 ± 0.37	3.11 ± 0.15

### Seasonal spatio-temporal evolution of PSB *C*. *okenii* and GSB *Chlorobium* spp. in the chemocline of Lake Cadagno

Total density of the photosynthetic community in the chemocline increased from 3 × 10^5^ cells mL^-1^ in June, to 5 × 10^5^ cells mL^-1^ at the end of the sampling season in October (Table 2). Specifically, peak density was observed in August, when cell density reached 1.4 × 10^5^ and 2.6 × 10^5^ cells mL^-1^ for PSB *C*. *okenii* and GSB *Chlorobium* spp., respectively, corresponding to 22% for *C*. *okenii* and 43% for *Chlorobium* spp., of all photosynthetic microorganisms measured by FCM in the chemocline. A marked difference between *C*. *okenii* and *Chlorobium* spp. was observed in October with a 8-fold reduction of *C*. *okenii* (1.8 ± 1.4 cells mL^-1^) and a 2-fold increase of *Chlorobium* spp. (51.2 cells mL^-1^ ± 14.3) compared to August.

**Table 2:** Seasonal cell density for PSB *C*. *okenii* and GSB *Chlorobium* spp. in the chemocline of Lake Cadagno chemocline determined by FCM.

Parameter measured	Cells	Month				
		June	July	August	September	October
Concentration of PSB *C*. *okenii*	10^4^ mL^-1^	5.3±2.9 (10)	11.2±5.4 (16)	14.0±3.4 (8)	6.4±2.9 (3)	1.8±1.4 (4)
Concentration of GSB *Chlorobium* spp.	10^4^ mL^-1^	9.1±5.1 (18)	24±8.1 (33)	26±7.0 (20)	15.3±3.7 (20)	51.2±14.3 (6)

Shown are arithmetic mean values ± standard deviation, followed by the number of samples in brackets.

### Temporal variation in cell complexity of PSB *C*. *okenii* and GSB *Chlorobium* spp. in the chemocline of Lake Cadagno

**Fig 7:**
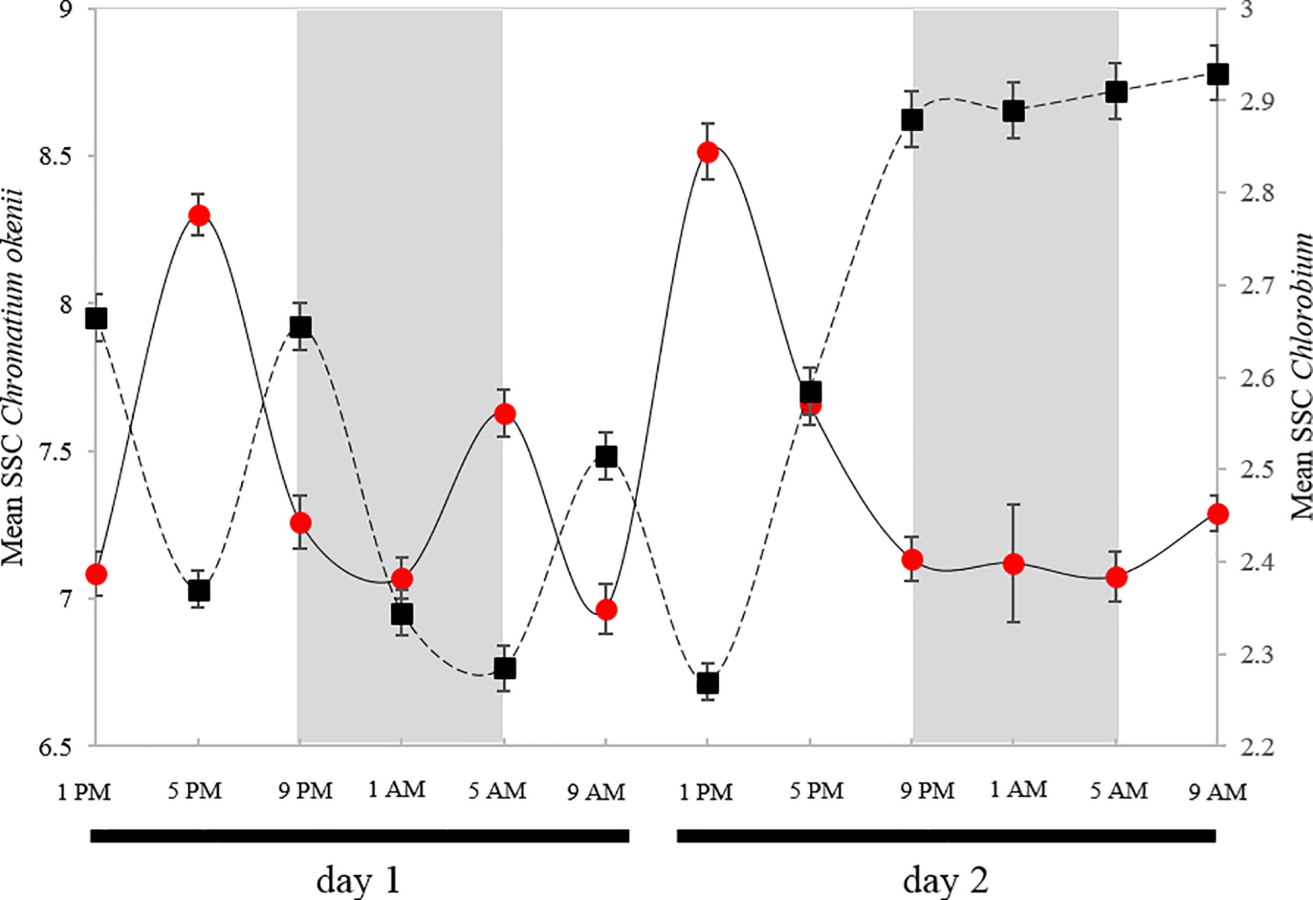
*In situ* cellular complexity variation of PSB C. okenii and GSB Chlorobium *spp*. SSC mean values (× 10^5^) and 95% CIs for total cells of PSB *C*. *okenii* (black squares, dashed lines) and GSB *Chlorobium* spp. (red circles, solid lines) populations. Analysis was done over two consecutive days (4 and 5 August 2015) at the depth with maximal cellular density (PSB and GSB combined). (Day1: 1PM, 12.8 m; 5PM, 12.2 m; 9 PM, 12.1 m; 1AM, 12.1 m; 5AM, 13.1 m; 9AM, 12.8 m. Day 2: 1PM, 12.1 m; 5PM, 12.5 m; 9PM, 12.6 m; 1AM, 12.9 m; 5 AM, 12.9 m; 9 AM, 12.7 m). Lines represent tentative interpolations between sampling occasions.

During the first 24-hours period of measurement, mean SSC of *C*. *okenii* and *Chlorobium* spp. cells, respectively, showed a regularly alternating pattern which was independent of the day/night period (Fig 7). Specifically, *C*. *okenii*’s cellular complexity exhibited maximal SSC values simultaneously with minimal values of *Chlorobium* spp. and vice-versa. However, after 28 hours, the SSC signal stabilized at relatively high values for *C*. *okenii*’s cells (SSC mean value between 8.5 × 10^5^ and 9 × 10^5^), whereas the SSC values of *Chlorobium* spp remained low (SSC mean value around 2.5 × 10^5^). Explorative statistics based on 95%-confidence intervals revealed statistically significant differences in SSC between both groups.

## Discussion

The chemocline of the meromictic Lake Cadagno harbours a complex microbial community in which phototrophic sulfur bacteria play key roles in various biogeochemical cycles including the sulfur and carbon cycle [41]. In this study, we applied FCM for the identification and characterization of phototrophic sulfur bacteria under both laboratory conditions and in their natural environment. A similar approach was previously presented by Casamayor and colleagues in Lake Vilar [30], which is also meromictic with a sulfide-rich monimolimnion. However, the phototrophic sulfur bacteria community differs greatly between the two systems. In particular, PSB *C*. *okenii* present in Lake Cadagno is known to play fundamental ecological roles as major contributor in inorganic carbon fixation [15] which can be studied due to its large cellular size using FCM. Our FCM analysis clearly differentiate PSB from GSB. Moreover, our FCM quantifications were clearly validated by FISH quantification (Table 1).

The response to sulfide oxidation during photosynthetic reaction was conducted for the most relevant PSB and GSB populations present in Lake Cadagno, i.e large-celled PSB *C*. *okenii*, small-celled *Candidatus* “T. syntrophicum” strain Cad16^T^ and GSB *C*. *phaeobacteroides*. PSB large-celled *C*. *okenii* and small-celled *Candidatus* “T. syntrophicum” strain Cad16^T^ alter significantly their cellular complexity in response to light-dependent H_2_S oxidation and accumulation of SGBs [30,42], as evidenced by SSC (Fig 1). Congruent with this observation, no sulfide oxidation and no SSC variation were measured under dark incubation. The measured ΔSSC variation determined in the incubation and the relative maximal SSC value was the result of SGBs accumulation, and both values were dependent on the starting sulfide concentration. In this experiment, sulfide concentrations used for incubation were in the range measured in the monimolimnion of Lake Cadagno. Our findings, correlating the sulfide oxidation and the intracellular complexity, thus corroborate those from meromictic Lake Vilar [30].

Moreover, in our study H_2_S consumption and intracellular SGBs storage in PSB *C*. *okenii* correlated with increasing signal of single or double stranded DNA and RNA detected by Sybr Green staining (FL1 in FCM) suggesting higher metabolic activity under light incubation (Fig 3). Consequently, we cannot exclude some influence of accumulating nucleic acid material in SSC variation [31]. Phototrophic sulfur bacteria are known to store also other inclusion bodies than SGBs such as glycogen and PHB that might influence the SSC signal [10]. However, the changes in cellular complexity due to metabolic activity did not influence the cellular size (represented by FSC) which remained constant during short period of photosynthetic activity. Together this data indicates that the photosynthetic process occurring in PSB stimulated intracellular physiological alteration producing inclusion bodies and DNA/RNA without affecting cell size. In contrast to PSB, GSB *C*. *pheobacteroides* oxidized H_2_S secreting SGBs into the surrounding environment without inducing alterations of the internal structure of cells [8,9,24,43,44]. GSB are obligate photoautotrophs [45] and require light for metabolic activity and production of inclusion bodies [10]. Interestingly, when comparing SSC values of *C*. *phaeobacteroides* previously incubated in light (Fig 2D) and dark (Fig 2E) we observed a higher cellular complexity independent of SGBs in light. In this case, the variation in intracellular complexity was probably the consequence of variations in glycogen, PHB and other inclusion bodies detected by SSC.

We investigated population dynamics of photosynthetic sulfur bacteria in the chemocline of Lake Cadagno. Both daily and seasonal trends showed that anoxygenic phototrophic PSB *C*. *okenii* and GSB *Chlorobium* spp. co-occured with maximal cell densities at the same depth. This result partly contrasts PSB and GSB occupancy in Lake Vilar, where higher density of PSB over GSB was found at 4.65 meters depth and at 5 meters depth the opposite trend was found [30]. However the apparent coexistence of PSB and GSB in the same ecological niche was already reported from other meromictic lakes [46,47] and in benthic systems [48]. These studies and ours demonstrate that coexistence of microbial species with similar metabolisms and sharing the same ecological niche might not be unusual in nature.

FCM has been extensively applied in the field of aquatic microbial ecology for the detection of photosynthetic organisms in their natural habitat [28–30]. In Lake Cadagno the peak FCM intensity of the chlorophyll signal coincided with maximal turbidity in the chemocline (Fig 4). These findings are in accordance with patterns of chlorophyll *a*, bacteriochlorophyll-*a* and bacteriochlorophyll-*e* measured during other studies in the chemocline of Lake Cadagno [20,24,40]. In the oxic mixolimnion, the red fluorescence signal was probably mainly due to the presence of picocyanobacteria such as *Synechococcus* spp. [40]. While in the anoxic monimolimnion, the FCM data presented a large fraction of red-fluorescence signal that matched with GSB density also in the zone with limited light intensity [20,24]. These findings suggest that in the absence of light GSB *Chlorobium clathratiforme* may obtain energy from the fermentation of polyglucose [24].

Due to their abundance in the chemocline of Lake Cadagno, PSB *C*. *okenii* and GSB *Chlorobium* spp. were rapidly discriminated and characterized by FCM. In Fig 5A, peaks 2 and 3 in the red fluorescent histogram revealed the presence of two photosynthetic populations with diverse chlorophyll signatures. BChl *a* and BChl *e* as the main antenna pigments of *C*. *okenii* and *Chlorobium* spp, respectively, could explain this difference [7]. Fluorescence *in situ* hybridization (FISH) confirmed FCM quantification results and validated its application for rapid quantification of the two dominant anoxygenic photosynthetic populations in the chemocline of Lake Cadagno (Table 1).

Additionally, SSC vs FSC in FCM scatterplot presented other cells than *Chlorobium* spp. and PSB *C*. *okenii* (Fig 5B). Small-celled PSB belonging to the genus *Lamprocystis* [13], *Thiocystis* [49] and *Thiodictyon* [50] are known to inhabit the Lake Cadagno chemocline. These populations were detected and quantified using FISH (S1 Table). However, although small-celled PSB presented a different SSC vs FSC scatterplot than *C*. *okenii* and *Chlorobium* spp. (S3 Fig), their detection using FCM is challenging due to their relatively low abundance (< 1% of all cells) and the dominant signature of highly concentrated *Chlorobium* spp. and the large cell size of *C*. *okenii*. To overcome this shortcoming FCM in combination with FISH was proposed for analyzing mixed microbial populations [51] and could be useful for small-celled PSB detection and quantification in Lake Cadagno.

Interestingly, compared to past studies investigating anoxygenic phototrophs population dynamic in Lake Cadagno [22,23], our quantitative data indicated a clear decrease in the concentration of the GSB community in the chemocline of Lake Cadagno since 2007 when GSB *C*. *clathratiforme* (as well as total photosynthetic community) reached 10^7^ cells mL^-1^ [20]. In contrast, our data (both FCM and FISH), estimated GSB cell density (as well as total photosynthetic community) as 10^5^ cells mL^-1^. Therefore, the overall decrease of the photosynthetic community could indicate a reduction in GSB *Chlorobium* spp. population size in this period. The increasing abundance of *Chlorobium* spp. observed after year 2000 may be the consequence of chemocline disruption with alteration in environmental niche [22]. Our data indicate a slow recovery of the situation which was present before year 2000.

In order to gain insight into the interaction between PSB and GSB in the chemocline of Lake Cadagno, changes in SSC values were monitored at high temporal resolution during a 48h-period (Fig 7). Although no strong variations in median SSC were observed (as in the case of SGBs accumulation in Fig 1), for both PSB and GSB populations the mean with 95% confidence intervals of SSC values showed an oscillating pattern in the chemocline. Interestingly, the respective patterns of PSB *C*. *okenii* and GSB *Chlorobium* spp. were regularly anticyclical. These oscillations were time-independent but apparently not linked to circadian rhythm mechanisms [52,53]. In particular, these patterns are at odds with those reported from Lake Cisó, which were synchronised with daylight hours [10,54]. Moreover, the fluctuating pattern revealed in our study suggests a plastic metabolic behaviour permitting adaptability and coexistence in natural habitats. In both PSB and GSB, the slight alteration in population complexity might be principally driven by alterations in nucleic acids (DNA or RNA) content in response to cellular metabolic activity, as observed for PSB *C*. *okenii* (Fig 3). This behaviour might probably be coordinated by inter-specific cell-cell communication between PSB and GSB [55]. During the second day, the oscillating pattern was replaced by a constant albeit synchronised (i.e. inverted) pattern of cellular complexity. These relationships between the metabolic activity of PSB and GSB could be the consequence of external environmental factors that influence interspecific responses. Furthermore, PSB *C*. *okenii* and GSB *Chlorobium* spp. coexistence could be one of the principal biological reason of bioconvection phenomenon recently described in Lake Cadagno chemocline [17]. The characterization of environmental drivers controlling these interlinked synchronous patterns of PSB and GSB metabolic activity warrants further studies.

## Conclusion

In this study, we proposed FCM as tool for fast detection and quantification of anoxygenic photosynthetic sulfur bacteria in the chemocline of meromictic Lake Cadagno. Anoxygenic photosynthetic PSB *C*. *okenii* and GSB *Chlorobium* spp. co-occur in the same ecological niche, sharing the same nutrients that may become limiting. Our FCM data revealed an unexpected interspecific equilibrium probably based on synchronous alternation of the metabolic activity of PSB and GSB populations. This process could be relevant for the dynamics and activity of the community of anoxygenic photosynthetic sulfur bacteria in the chemocline of Lake Cadagno and to understand the underlying mechanism supporting coexistence of these dominant taxa.

To further investigate these relationships and potential influence of one photosynthetic population on the other in their natural environment, we suggest the use of FCM in combination with single population sorting and supplementary metabolic analysis [56,57]. This procedure holds the potential to link structure variations determined through FCM SSC variations to physiological activity of single population at defined temporal intervals.

## Supporting information

S1 Fig Phase contrast microscopy (1000x magnification) image of anoxygenic phototrophic purple sulfur bacterium *Chromatium okenii* with presence of intracellular sulfur inclusion bodies (SGBs). (TIF)

S2 Fig Flow-cytometry size calibration kit (Molecular Probes, F-13838). The kit contains six suspension of unstained polystyrene microspheres (A = 1.0 μm-diameter, B = 2.0 μm-diameter, C = 4.0 μm-diameter, D = 6.0 μm-diameter, E = 10.0 μm-diameter, F = 15.0 μm-diameter). The size of cells in an experimental sample can be estimated by comparing the FSC signals with those of the reference microspheres.
(TIF)

S3 Fig Flow cytometry signature of (left) pure culture anoxygenic phototrophic green sulfur bacterium *Chlorobium clathratiforme* (Median FSC: 5’956.0 = 1.6 μm), (middle) pure culture anoxygenic phototrophic purple sulfur bacterium *Candidatus* “Thiodictyon syntrophicum” strain Cad16^T^ (Median FSC: 93'254.5 = 2 μm and (right) Lake Cadagno enrichment of anoxygenic phototrophic purple sulfur bacterium *Chromatium okenii* (Median FSC: 886’559.5 = 6.7 μm). (TIF)

S1 Table Cy3-labeled oligonucleotide probes used in this study for FISH counting. (TIF)

S2 Table FISH probes percentage for most relevant phototrophic sulfur bacteria in Lake Cadagno chemocline. (TIF)

## Abbreviations

PSB: purple sulfur bacteria
GSB: green sulfur bacteria
FCM: flow cytometry
FSC: forward scatter
SGB: sulfur globules
SSC: sideward scatter
FISH: fluorescent in situ hybridization
