# Supplementary figures and images for: Dynamic cellular complexity of anoxygenic phototrophic sulfur bacteria in the chemocline of meromictic Lake Cadagno
Source: PLoS One. 2017 Dec 15;12(12):e0189510. doi: 10.1371/journal.pone.0189510 (PMC5731995; doi:10.1371/journal.pone.0189510)

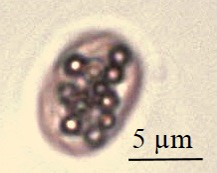

Supplement: S1 Fig — (TIF) [file pone.0189510.s001.tif]

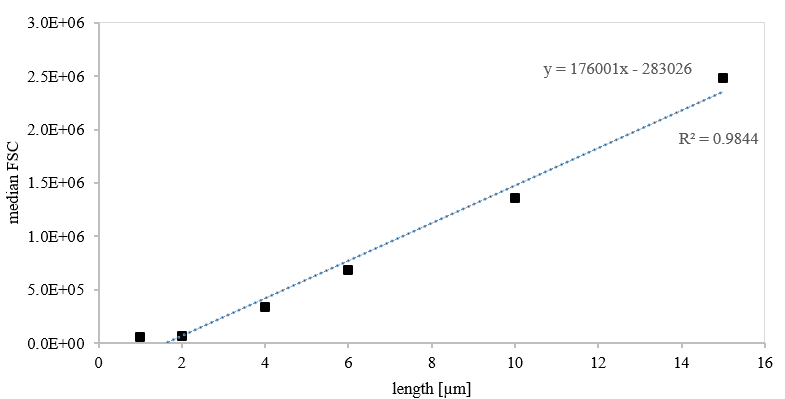

Supplement: S2 Fig — The kit contains six suspension of unstained polystyrene microspheres (A = 1.0 μm-diameter, B = 2.0 μm-diameter, C = 4.0 μm-diameter, D = 6.0 μm-diameter, E = 10.0 μm-diameter, F = 15.0 μm-diameter). The size of cells in an experimental sample can be estimated by comparing the FSC signals with those of the reference microspheres. (TIF) [file pone.0189510.s002.tif]

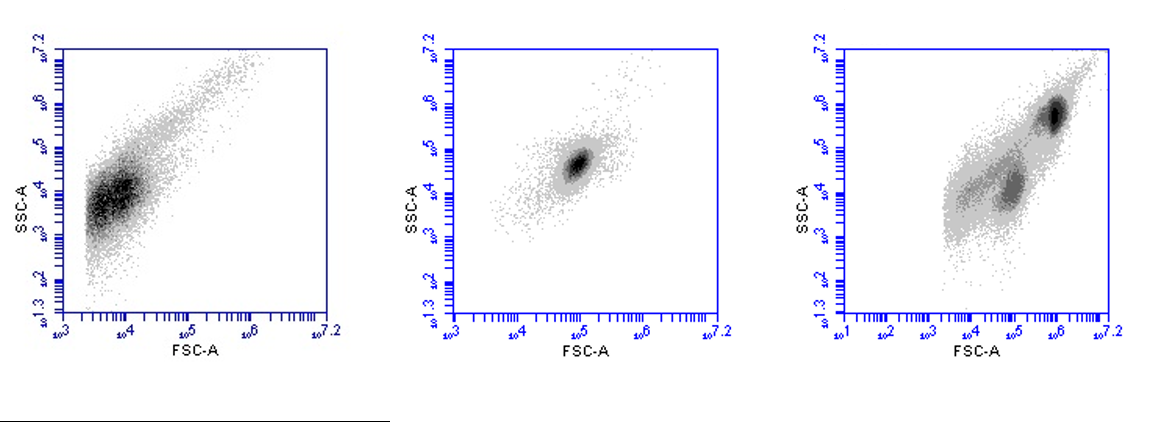

Supplement: S3 Fig — (TIF) [file pone.0189510.s003.tif]

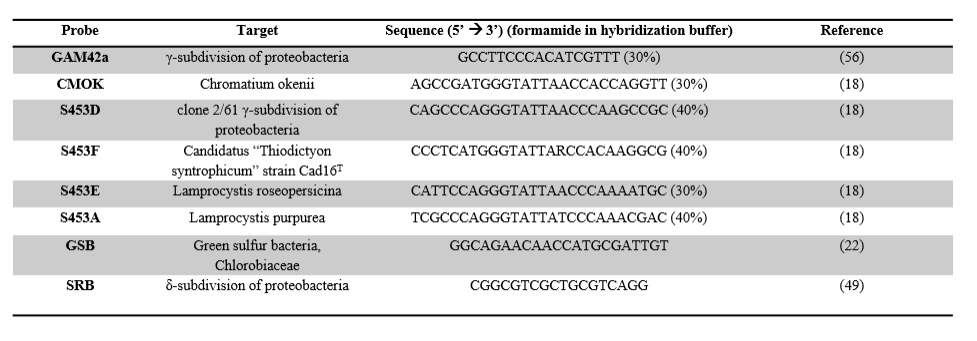

Supplement: S1 Table — (TIF) [file pone.0189510.s004.tif]

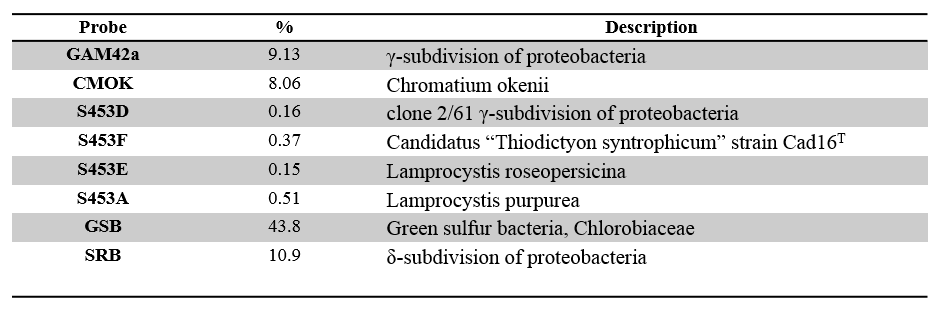

Supplement: S2 Table — (TIF) [file pone.0189510.s005.tif]
